# *Clostridium tyrobutyricum* occurrence in silages and cattle feed: Use of molecular and simulation data to optimize predictive models

**DOI:** 10.3389/fmicb.2023.1118646

**Published:** 2023-03-23

**Authors:** Martina Mosconi, Alessandra Fontana, Mireya Viviana Belloso Daza, Daniela Bassi, Antonio Gallo

**Affiliations:** ^1^Dipartimento di Scienze animali, della nutrizione e degli alimenti (DiANA), Università Cattolica del Sacro Cuore, Piacenza, Italy; ^2^Dipartimento di Scienze e Tecnologie Alimentari per una Filiera Agro-Alimentare Sostenibile (DISTAS), Università Cattolica del Sacro Cuore, Piacenza, Italy

**Keywords:** *Clostridium tyrobutyricum*, late-blowing defect, predictive modeling, silage, real-time PCR, farm residues, dairy cows

## Abstract

**Introduction:**

Poor quality silage can derive from the presence of deleterious microorganisms such as clostridia. Their dissemination along the food chain, especially in milk, causes issues such as the cheese late-blowing defect, particularly triggered by *Clostridium tyrobutyricum*. The scope of our study was to determine the *C. tyrobutyricum* occurrence in three different farms across four time periods in relation to the animal diets, specifically the Total Mixed Ration (TMR), by using real-time PCR.

**Methods:**

For this purpose, molecular-derived data were exploited to optimize a predictive model that simulated the farm conditions favoring the growth of butyric acid bacteria such as *C. tyrobutyricum*.

**Results:**

Our results showed that the originally utilized predictive model strongly underestimated the growth of *C. tyrobutyricum* in comparison to the molecular data. At the same time, our findings uncovered an additional source of contamination in the TMR related to silage and dietary residues that represent a reservoir of microbial contamination during successive TMR preparation. Based on these findings, the optimization of the model parameters such as growth rate range and the inclusion of the residues in the model, allowed a more accurate prediction of the contamination levels. Therefore, this study revealed that proper hygiene practices such as the removal of silage and TMR residues within the farm environment is essential to control the contamination by *C. tyrobutyricum* and avoid food waste and economic losses.

## 1. Introduction

*Clostridium tyrobutyricum* is considered the main responsible of the late-blowing defect, namely off-flavors and excessive gas formation in hard cheese (Nishihara et al., 2014; Bassi et al., 2015; Storari et al., 2015). This bacterial species belongs to the butyric acid bacteria (BAB), a group of microorganisms that can convert lactic acid into butyric acid, hydrogen, and carbon dioxide, at low pH (Vissers et al., 2007a). It has been stated that the occurrence of BAB spores in milk derives from the farm environment, as they are naturally present in soil and animal feces (Vissers et al., 2006; Murphy et al., 2016). To avoid late-blowing defects, previous works reported different strategies: (i) reduction of the contamination level of milk directly at the farm, (ii) the use of nitrates and lysozyme during the process of cheese production, and (iii) the use of bactofugation to remove spores up to 99% during cheese production. In addition, feed quality, cattle-house hygiene, and milking practices, are also key factors in the milk contamination by BAB (Zucali et al., 2015; Calamari et al., 2018). To identify the main factors related to the presence of BAB in milk at the farm level, Vissers et al. (2006) proposed a simulation model. The model assessed that BAB presence was mainly due to the microbial contamination of silages, in which BAB can be present in high counts due to the physico-chemical conditions that are optimal for their growth and development (Gallo et al., 2016a). Ensiling is a preservation method used for moist crops and is based on naturally occurring lactic acid bacteria (LAB) fermentation under anaerobic conditions, where the reduced pH inhibits the growth of deleterious microorganisms (Zheng et al., 2017). However, previous studies reported that incorrect ensiling practices could favor the penetration of air into the ensiled mass, thus negatively affecting the quality of the ensiled products (Driehuis et al., 2001; Gallo et al., 2016b), and enhancing the development of spore-formers like clostridia (Vissers et al., 2007b; Zucali et al., 2015). Considering the silage mass, the apical and lateral areas are those which are most affected by these negative phenomena as the practice of sealing and pressing the bunker in these areas is more difficult (Vissers, 2009; Borreani and Tabacco, 2010; Gallo et al., 2016c).

In Italy, since many years, it is a common practice to feed large dairy herds with a total mixed ration (TMR) that is obtained by mixing forages (silage and hay), by-products, concentrates, minerals, vitamins, and additives in a unique solution. From the TMR, animals can obtain a uniform and nutritionally complete diet (Bueno et al., 2020). It is worth highlighting that each farm has its own typical TMR composition, which, particularly in terms of silage composition, can determine a specific level of BAB, particularly *C. tyrobutyricum*, contamination that cannot be compared among farms; it is the quantity and quality of raw materials included in the TMR that determine the level of microbial load (Driehuis, 2013). To simulate microbial growth in TMR, a previous study used a predictive tool based on the Baranyi growth model (Baranyi and Roberts, 1994), that included a maximum attainable contamination level (C∞), which could be relevant for the microbial growth estimation (Vissers et al., 2006). Moreover, Vissers and colleagues estimated the growth rate using the gamma concept of Zwietering et al. (1996), as the effects of temperature and pH can be separated.

The aim of our study was to determine the major sources of *C. tyrobutyricum* contamination at the farm level in relation to the silages and animal diets, by using a recent validated real-time PCR (Bassi et al., 2013; Arnaboldi et al., 2021) and exploit molecular detection data to optimize a predictive model that simulated the farm conditions favoring the growth of *C. tyrobutyricum* in feeds and diet. Particularly, we choose to apply and optimize the Vissers’ model to predict *C. tyrobutyricum* contamination within the TMR in three different farms, to collect preliminary data useful for subsequent model validation.

## 2. Materials and methods

### 2.1. Sampling procedure

For this study, silages (corn silage, small grain silage, legume silage, wrapped bales), high moisture corn (HMC), hays (alfalfa hay, grass hay, polyphytic hay), wet co-products (tomato peelings, brewers), TMR, TMR and silage residues were collected and analyzed to evaluate the contamination of *C. tyrobutyricum*,as detailed below.

Samples were taken considering four different periods (January 2020, July 2020, March 2021, and December 2021), in three different farms (respectively Farm 1, Farm 2, and Farm 3) located in the Po Valley (Italy). In January 2020, July 2020, and March 2021, samples were taken in three different days for each matrix, to create a final sample pool. The silage and TMR residues were not collected during these samplings, as the model proposed by Vissers et al. (2006) did not incorporate them. However, as part of the model optimization process, we included the silage residues that remained in the bunker base and the TMR residues that remained in the mixing wagon and manger, which were sampled in December 2021. Regarding the sampling of silage, the outermost layer of the bunker front was removed, due to oxygen exposure. All the samples were promptly frozen at −20°C after withdrawal.

The parameters measured for each sample matrix were: TMR composition, time (*t,* time that had passed from the preparation of TMR until sampling), TMR temperature, quantification of residues (remains in the bunker base, mixing wagon, and cattle feeder), pH, and *C. tyrobutyricum* cells and spores contamination. The pH was measured with a pH meter (Crison Micro-pH, Barcelona, Spain) on an aqueous extract, which was obtained by diluting the fresh material in distilled water (1:3 ratio) and homogenized with a Stomacher blender (Seward Ltd., West Sussex, UK) for 3 min; the blend was then filtered through a medical non-woven gauze.

### 2.2. Total DNA extraction and real-time PCR for *Clostridium tyrobutyricum* quantification

Total DNA extraction was performed using Dneasy PowerLyzer PowerSoil kit (Qiagen, Hilden, Germany), according to the manufacturer’s instructions. Sample preparation for DNA purification of silages, HMC, hays, wet co-products, TMR, TMR residues, and silage residues was done as follows: 10 grams of each sample were added to 90 ml of physiological solution and processed (2 × 30 s at maximum speed) in a Stomacher apparatus (Biochek, Foster City, CA, United States) using filter bags. Of these homogenates, 1.0 ml was collected and centrifuged (13,000 × g, 10 min) and DNA extracted from the resulting pellets The first mechanical lysis was carried out using the FastPrep homogenizer (MP Biomedicals™, California, United States) for 45 s at 4.5 m/s. The extracted DNA was quantified using the Qubit® Quantitation Platform and then stored at −20°C to be used for *C. tyrobutyricum* detection using real-time PCR assays.

TaqMan real-time PCR was performed using the StepOnePlus™ Real-Time PCR System (Applied Biosystems Japan, Tokyo, Japan). Standard curve and thermal profiles were carried out according to the protocol developed by Bassi et al. (2013). *C. tyrobutyricum* UC7086 (Università Cattolica culture collection) was used to construct a five point-based standard calibration curve using triplicates set of 10-fold dilutions of genomic DNA extracted from a 3×10^9^ GE/mL, Genome Equivalent *C. tyrobutyricum* UC7086 pure culture (DNA concentrated 1.2 ng); calculation was made taking in consideration the mean *C. tyrobutyricum* ATCC25755^T^ genome length of 3,053 Mb. The five points used for the curve ranged from 3.6 × 10^5^ to 3.6 × 10^1^ CFU/ml. The real-time reaction was set using a total volume of 20 μl, containing 10 μl of TaqMan reaction mix (Promega), 0.4 μM of each primer, 0.1 μM of *pta* probe and 5 μl of DNA. All the unknown samples were processed in triplicate. In each run, negative and positive controls were included.

Real-time results were analyzed using the Second Derivative Maximum Method and Microsoft® Excel (Microsoft Italia, Milano, Italy) was used to perform the statistical data analyses. Means and standard deviations of Log-based *C. tyrobutyricum* concentrations were calculated for each feed sample.

### 2.3. Modeling of *Clostridium tyrobutyricum* contamination

The *C. tyrobutyricum* contamination was estimated by using the model proposed by Vissers et al. (2006). In this model, Vissers reported the sources of contamination corresponding to raw materials consisting of three stocks, being soil, silage, and other feeds. For microbial growth, this model considers Baranyi growth model (Baranyi and Roberts, 1994), whereas the growth rate was estimated using the gamma concept of Zwietering et al. (1996). Regarding the seven-unit operations in the proposed model, only the first two were analyzed in the present study to determinate *C. tyrobutyricum* contamination in TMR. Soil, presented as a source of contamination, was in the fourth-unit operation and thus it was not considered in this study.

The parameters required by the model to predict the *C. tyrobutyricum* contamination of the TMR at time *t* are divided into two classes: measured parameters, which are associated with variability due to possible errors, and constant parameters, retrieved from the relevant literature. The time *t*, as defined by Vissers et al. (2006), refers to the storage time that goes from the creation of the TMR until its ingestion by the animals. During this time, BAB growth is dependent on temperature, pH, and nutrient availability. In our case, time *t* represents the time interval between the preparation of the TMR and its sampling.

The measured parameters include the concentration of *C. tyrobutyricum* in each analyzed matrix (CFU/g) with the respective percentage of inclusion in the TMR, the time between TMR preparation and sample collection, the lag time (λ) representing the initial stasis phase preceding microbial growth; the temperature of the ration (°C); and the pH of the ration (dmnl). The constant parameters are represented by the growth characteristics of BAB and the maximum achievable concentration (Log CFU/g) of BAB. Briefly, in the Vissers model (here called Original Vissers Model or OVm) all food matrices used in TMR are included considering the relative percentage of each component within the diet to calculate the theoretical TMR concentration at time 0 according to the following equation (Eq. 1):

(1) $$\begin{array}{l} C_{TMR\left(0\right)}=F_{silage1}*C_{silage1}+F_{silage2}*C_{silage2}+\ldots +\\ \left(1\_\left(F_{silage1}+F_{silage2}+\ldots \right)\right)*C_{otherfeed} \end{array}$$

Eq. 1: Theoretical TMR concentration of *C. tyrobutyricum* at time 0.

Where C_TMR_(0) is the contamination of ration at time 0 (CFU/g); *F*_silage1_ is the percentage of silage 1 within the ration (%); C_silage1_ is the contamination of silage 1 (CFU/g); F_silage2_ is the percentage of silage 2 within the ration (%); C_silage2_ is the contamination of silage 2 (CFU/g), and C_otherfeed_ is the contamination of the other feeds in the ration (CFU/g).

In OVm, the formula for estimating the concentration at time *t* is also given, as indicated in the equation below (Eq. 2):

(2) $$C_{TMR\left(t\right)}=\exp\left(\begin{array}{l} \ln\left[C_{ration}\left(0\right)\right]+\mu *A_{N}\left(t\right)-\\ \ln\;\left(1+\left(e^{\mu *A_{N}\left(t\right)}*t\right)/\left(e^{lnC\infty /C_{ration}\left(0\right)}\;\right)\right) \end{array}\right)$$

Eq. 2: Theoretical TMR concentration of *C. tyrobutyricum* at time *t.*

Where C_TMR_ (*t*) is the ration concentration at time *t* (CFU/g), *μ* is the growth rate (h^−1^), A_N_(*t*) is the multiplication during time (*h*), *t* is the time from the preparation of the ration to the moment it is ingested-sampled, and C_∞_ is the maximum attainable contamination level (CFU/g).

The purpose of the model was to predict *C. tyrobutyricum* contamination values of the TMR close to those measured by real-time PCR.

Considering the possible errors in the measurement of the data, along with the range in which these could vary, a second model was developed, the so-called Expanded Vissers model (EVm). In EVm, the microbial growth rate was modified including a wide range of values, from 0.12 h^−1^ (Vissers et al., 2006) to 0.43 h^−1^ (Ruusunen et al., 2012). In the EVm, the optimization of the parameters into the fixed range was performed using the Excel Optimizer Solver (Microsoft Office Professional Plus 2010®, Microsoft Corporation, WA, United States). The conditions under which the optimizer operated were: linear SIMPLEX resolution method, convergence 0.000001, and mutilation rate 0.075. The measured parameters entered the model were optimized according to the set range (±10%). The optimizer allowed to determine the optimal solution of a linear programming problem.

The silage and TMR residues were not considered in the previous models, therefore a third model, the expanded Vissers model with residues (EVmR), was integrated and tested for its ability to properly predict final *C. tyrobutyricum* contamination of diet. In particular, the residues concentration of *C. tyrobutyricum* at time *t* was predicted by adopting the approach already showed in equation 2, by opportunely substituting the specific terms. These residues (i.e., silage and TMR) were then added to summative eq. 1.

It is important to specify that among TMR residues, leftovers from the cattle feeder were not considered within the model, since the correct management practices in the farm suggest their removal before carrying out the new administration of the animal feed. Therefore, no TMR residues in the cattle feeder should be found in a well-operating farm (Amaral-Phillips, 2001).

## 3. Results

### 3.1. Molecular-based detection of *Clostridium tyrobutyricum* in feed and farm samples

The molecular method based on real-time PCR was used to determine the occurrence of *C. tyrobutyricum* in the matrix components of the three different farms object of the study (Table 1).

**Table 1:** Real-time PCR values of *Clostridium tyrobutyricum* contamination (CFU/mL) within the different matrices and in the TMR, across the three farms and the four different periods.

Matrix	January 2020	July 2020	March 2021	December 2021	Mean value
***Farm 1***					
*Corn silage*	<LOD*	<LOD	<LOD	<LOD	<LOD
*Other silage*	<LOD	<LOD	4.77 ± 2.70	4.77 ± 2.70	3.12 ± 2.37
*Alfalfa hay*	2.56 ± 1.87	N.A.	<LOD	N.A.	2.02 ± 1.32
***TMR***	6.71 ± 0.04	6.90 ± 0.06	6.63 ± 0.09	6.63 ± 0.09	6.72 ± 0.13
***Farm 2***					
*Corn silage*	2.50 ± 1.78	3.49 ± 1.75	2.44 ± 1.67	<LOD	2.48 ± 1.48
*Other silage*	<LOD	2.52 ± 0.92	<LOD	<LOD	1.74 ± 0.61
*Alfalfa hay*	<LOD	N.A.	<LOD	N.A.	<LOD
*Other hay*	N.A.	N.A.	<LOD	N.A.	<LOD
***TMR***	5.84 ± 0.05	5.51 ± 0.07	6.37 ± 0.03	5.34 ± 0.16	5.77 ± 0.42
***Farm 3***					
*Corn silage*	4.83 ± 0.24	5.18 ± 0.18	5.87 ± 0.02	<LOD	4.34 ± 1.78
*Other silage*	4.09 ± 0.54	6.08 ± 0.06	2.72 ± 1.09	<LOD	3.59 ± 1.86
*Other hay*	N.A.	N.A.	<LOD	N.A.	<LOD
*Wet co-product*	N.A.	N.A.	6.58 ± 2.07	N.A.	6.58 ± 2.07
***TMR***	5.95 ± 0.04	6.51 ± 0.01	7.20 ± 0.04	5.93 ± 0.18	6.40 ± 0.55

Log values are reported as means and standard deviations of three replicates for each analyzed matrix.

*LOD: limit of detection (1.5 Log CFU/mL). TMR (in bold) and TMR constituents (in italic). N.A.: matrix not available at the considered month.

Briefly, in all three farms, samples of TMR and related components were taken in four different periods, whereas residue samples (TMR and silage residues) were taken only during one period (December 2021). Average *C. tyrobutyricum* contamination levels of the feed constituting the TMR and the TMR and silage residues, are shown in Tables 1, 2.

**Table 2:** Real-time PCR values of *Clostridium tyrobutyricum* contamination (CFU/mL) in residues, across the three farms sampled in December 2021.

Matrix	Farm 1	Farm 2	Farm 3
Corn silage residues	6.62 ± 0.04	5.99 ± 0.15	6.62 ± 0.04
Other silage residues	3.69 ± 1.93	5.93 ± 0.10	3.69 ± 1.93
TMR residues	6.27 ± 0.52	5.94 ± 0.14	6.27 ± 0.52
Mixer wagon residues	5.84 ± 0.15	6.02 ± 0.20	5.84 ± 0.15

Regarding Farm 1, for the category *corn silage*, values below the LOD were detected in all four periods. For the category *other silage*, contamination of 4.8 Log CFU/mL was detected in March and December 2021, whereas in Januaryand July 2020, contamination below the LOD was determined. The analysis of *alfalfa hay* was possible in the winter period only, due to farm availability of this matrix: in January 2020 it showed a mean value of 2 Log CFU/mL. Considering TMR, an average of 6.7 Log CFU/mL was detected along the four periods.

In Farm 2, with regards to *corn silage*, an increase of 4.8 Log was found compared to farm 1. For *other silage*, a concentration of 2.5 Log CFU/mL was estimated in July 2020 only. As in the case of Farm 1, alfalfa hay was sampled in the winter period only with values below the LOD. An additional category was sampled in Farm 2, namely *other hay*, where values below the LOD were presented. TMR showed instead an average value of 5.8 Log CFU/mL throughout all considered periods.

In the case of Farm 3, contaminations across the four periods were highly variable for two categories: *corn silage* and *other silage* (Table 1). Specifically, when considering March and December 2021, an approximately 6 Log decrease was observed (from 5.9 Log CFU/mL to values below the LOD) for corn silage. Regarding *other silage*, the same trend was evidenced from July 2020 to December 2021. Categories *other hay* and *wet co-products* were sampled in March 2021 with values below the LOD and 6.6 Log CFU/mL, respectively. For TMR, an average value of 6.4 Log CFU/mL was determined, with highest counts in March 2021.

In December 2021, additional samples were collected from the residues of silage (both corn and small grain silage) and TMR (both from the mixing wagon and the manger leftovers). On all farms, these residues amounted to approximately 100 kg at each sampling point and had contamination values in the range of 3.7–6.6 Log CFU/mL (Table 2).

The comparison between *C. tyrobutyricum* contamination values within the silage and its residues after 24 h from de-ensiling, showed a higher contamination within the residues than the silages not yet de-ensiled. As a further confirmation of this finding, the sampling of the TMR residues that remain in the mixer wagon and in the manger for 24 h, showed a similar trend, with higher level of contamination within the residues than fresh TMR.

### 3.2. Modeling prediction of *Clostridium tyrobutyricum* using three different prediction models

**Figure 1:**
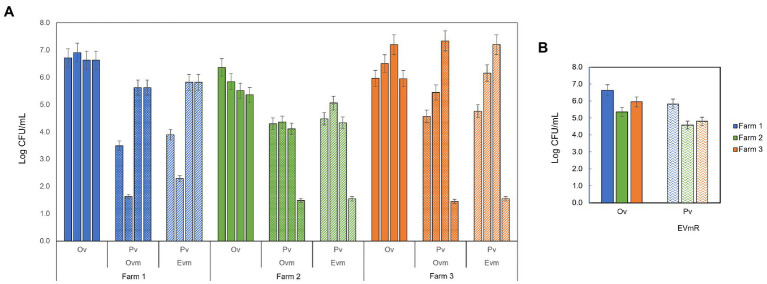
Comparison of *Clostridium tyrobutyricum* contamination levels (Log CFU/mL) observed experimentally (Ov) in the TMR, across the four time periods, for farm 1 (solid blue), farm 2 (solid green), and farm 3 (solid orange), and those predicted (Pv) in farm 1 (striped blue), farm 2 (striped green), and farm 3 (striped orange), by using the 3 models: **(A)** original Vissers model (OVm) and expanded Vissers model (EVm), and **(B)** expanded Vissers model with residues (EVmR).

A comparison between the contamination levels of *C. tyrobutyricum* detected in TMR by molecular approach and the prediction-based values from the applied models, are shown in Figure 1.

Taking a closer look at the predicted values in Farm 1, Ovm strongly underestimated observed values for all periods, whereas Evm reached values closer to the real-time PCR concentrations for March and December 2021 (i.e., differences below 1 Log CFU/mL). The same prediction was achieved by the EvmR.

The Ovm derived predictions in Farm 2 strongly differed from observed values in all periods. The Evm showed similar underestimations except for July 2020, where the difference with the observed value was below 1 Log. The EvmR was able to predict 4.59 Log CFU/mL in comparison to the observed value of 5.36 Log CFU/mL.

In contrast, in Farm 3 the Ovm successfully estimated contamination values in March 2021 and Evm was able to estimate accurately in July 2020 and March 2021. The predictions computed for December 2021 using Ovm and Evm were underestimated by almost 4.5 Log; instead, EvmR improved the prediction with a difference between observed and predicted value of 1.2 Log.

A comparison between the contamination levels of *C. tyrobutyricum* detected in TMR by molecular approach and the prediction-based values from the applied models, are shown in Figure 1.

Taking a closer look at the predicted values in Farm 1, OVm strongly underestimated observed values for all periods, whereas EVm reached values closer to the real-time PCR concentrations for March and December 2021 (i.e., differences below 1 Log CFU/mL). The same prediction was achieved by the EVmR.

The OVm derived predictions in Farm 2 strongly differed from observed values in all periods. The EVm showed similar underestimations except for July 2020, where the difference with the observed value was below 1 Log. The EVmR was able to predict 4.59 Log CFU/mL in comparison to the observed value of 5.36 Log CFU/mL.

In contrast, in Farm 3 the OVm successfully estimated contamination values in March 2021 and EVm was able to estimate accurately in July 2020 and March 2021. The predictions computed for December 2021 using OVm and EVm were underestimated by almost 4.5 Log; instead, EVmR improved the prediction with a difference between observed and predicted value of 1.2 Log.

## 4. Discussion

Poor quality silage is defined by the presence of butyric acid and ammonia nitrogen, highly related to clostridial activity and low concentrations of LAB (Zheng et al., 2017). Certainly, the presence of clostridia in raw materials implies their persistence throughout the food chain, especially in dairy products. In fact, *C. tyrobutyricum* is considered the main cause of late-blowing defect in hard cheeses (Vissers, 2009; Borreani and Tabacco, 2010; Gallo et al., 2016c).

The study focused on comparing, within each individual farm, the TMR contamination by *C. tyrobutyricum* measured experimentally by molecular methods, with the TMR contamination estimated by the application of predictive models. The *C. tyrobutyricum* quantification obtained from the molecular-based approach by real-time PCR, were used to validate the original model (OVm) presented by Vissers et al. (2006), based on the detection of BAB (e.g., *C. tyrobutyricum*) in milk at farm level. Next, based on these results, we developed further modeling approaches to fit observed data. In particular, OVm explained that the presence of BAB was mainly due to the microbial contamination of silage, in which BAB found optimal physico-chemical conditions for their growth and development (Gallo et al., 2016a). However, in this case, the quantification of the contamination of this microorganism was performed with culture-based techniques (MPN method and plate counts) that have generally lower sensitivity toward the determination of CFU/mL in a sample and that determine the total amount of clostridia spores. Differently, in this study, the use of molecular techniques such as real-time PCR ensured a highly sensitive detection of *C. tyrobutyricum*, increasing the detection and resolution capacity in complex matrices such as silage. Our results showed a sensitive limit of detection (1.5 Log CFU/mL), in line with a previous study, in which a multiplex real-time PCR was developed to detect *Clostridium* species in cheese with a detection limit of ~ 1 Log CFU/mL (Şahiner et al., 2022). In addition, data obtained in our validation study (Arnaboldi et al., 2021) showed that MPN results sometimes overestimated or underestimated the real clostridia amount in the samples and that the difference between MPN and real-time PCR was related to the specificity of the molecular method only quantifying *C. tyrobutyricum* DNA. The high sensitivity of the molecular-based method is further appreciated when compared to the predicted values of OVm, which overall underestimated the contamination values in all farms (Figure 1). Following this, we optimized the OVm by changing the growth rate parameter to a range of values used in previous studies (Vissers et al., 2006; Ruusunen et al., 2012), obtaining a model here called Extended Vissers model, EVm. Considering EVm, the growth rate variation intervals of ±10% allowed the model to identify the most accurate values, facilitating the recognition of possible detection errors. Indeed, the EVm increased the prediction accuracy in 50% of the considered periods in all the three farms.

Generally, TMR had higher measured contamination levels than the ones found in each of the TMR constituents (differences of 1 to 5 Log CFU/mL). Thus, we assumed that an additional source of contamination could be investigated extensively in the farm environment. We hypothesized that a possible source of microbial inoculum could be represented by the TMR residues remaining after 24 h in the mixer wagon and silage residues remaining at the base of the bunker following de-ensiling and collected during the subsequent preparation of the TMR.

It has been highlighted that an increased concentration of BAB spores is related with the aerobic instability of silages (Vissers et al., 2007a; Driehuis, 2013; Gallo et al., 2016a). Specifically, high concentrations of BAB spores were reported to be present in samples with high signs of aerobic spoilage, generally located in the upper layer of the bunker (~ 50 cm depth) and in portions of the bunker with low density, which can therefore be easily infiltrated by oxygen (Vissers et al., 2007a). De-ensiled material and TMR residues that remain at the base of the bunker and inside the mixing wagon for 24 h, putatively underwent microbial aerobic metabolism that might have induced oxygen depletion and short-chain fatty acids production, creating the proper conditions for *C. tyrobutyricum* growth. Moreover, in the same study, it was stated that the presence of corn and other silages in TMR could carry higher concentrations of BAB. In fact, inside the corn silage bunker, the contamination levels vary widely based on the portion considered. Despite the highly contaminated portions represented a low percentage incidence in the bunker (from 1 to 10%; Vissers et al., 2007b), they were an important source of BAB in the TMR. This could explain the importance of silage and TMR residues within the *C. tyrobutyricum* contamination cycle, which, although low in percentage incidence in the TMR composition, represents a non-negligible source of contamination (Vissers et al., 2007b), they were an important source of BAB in the TMR. This could explain the importance of silage and TMR residues within the *C. tyrobutyricum* contamination cycle, which, although low in percentage incidence in the TMR composition, represents a non-negligible source of contamination.

Since molecular-based analysis showed that residues reported a higher contamination level than TMR after 24 h, both silage and TMR residues were included in the EVm as additional TMR component, thus obtaining what has been defined EVmR. The use of this model, compared to the previous ones, allowed a more accurate prediction of *C. tyrobutyricum* contamination in the TMR, getting closer to the molecular measurements. Despite the consideration of only one sampling point, as an exploratory investigation, this implemented model was able to predict the importance of silage and TMR residues that act as an additional source of microbial inoculum during subsequent TMR preparation. Therefore, in addition to the correct management of the bunker with the removal of the de-ensiled material, a suitable cleaning of the mixing wagon appears to be an important factor to decrease *C. tyrobutyricum* contamination at farm and milk levels. This study may be preliminary to other research both regarding the environmental transmission of *C. tyrobutyricum* and considering seven-unit operations presented in the model proposed by Vissers et al. (2006).

Focusing on the microbiological perspective only, the high contamination level of *C. tyrobutyricum* found in the TMR and silage residues could be putatively related to a low presence of LAB in the silage, which are known to induce a rapid carbohydrates fermentation and thus a pH decrease by accumulating lactic acid. Acidification has indeed an important role in avoiding undesirable secondary fermentation by clostridial populations. Additionally, even if autochthonous LAB of forages are present, the low initial counts may not be sufficient to reduce the pH rapidly enough to counteract clostridial growth (Zheng et al., 2017). Moreover, the longer residence time of the residues in the mixer wagon and at the base of the bunker, cause a decrease of the available water-soluble-carbohydrates necessary for LAB maintenance, allowing secondary deleterious fermentation processes like butyric acid fermentation to occur (Rossi and Dellaglio, 2007).

## 5. Conclusion

This study implemented a prediction model able to estimate the contamination levels of *C. tyrobutyricum* in silage and TMR, through data obtained from the highly sensitive molecular-based approach by real-time PCR on a single microbial species that is the most responsible of the late-blowing defect in hard cheese.

The introduction of a growth rate interval for *C. tyrobutyricum*, along with the first time evaluation of the TMR and silage residues (even if analyzed in one farm only), allowed us to optimize the model with an additional index for a more reliable prediction of *C. tyrobutyricum* contamination in cattle feed. Our implemented model indicates that farm residues represent an additional reservoir of *C. tyrobutyricum* contamination during successive TMR preparation.

Therefore, a correct management of the bunker with the removal of the de-ensiled material, along with a suitable cleaning of the mixing wagon, appear to be important factors to limit *C. tyrobutyricum* contamination. However, these statements deserve future investigations, on a large number of dairy farms, to be validated and to define proper management practices to counteract the risk of contamination from silage and TMR residues.

## Data availability statement

The original contributions presented in the study are included in the article/supplementary material, further inquiries can be directed to the corresponding author.

## Author contributions

MM, AF, and MB: methodology, investigation, data curation, writing – original draft, and writing – review and editing. DB and AG: conceptualization, writing – original draft, data curation, writing – review and editing, and supervision. All authors contributed to the article and approved the submitted version.

## Funding

This work was supported by “Fondazione Romeo ed. Enrica Invernizzi” as part of “Safety of Silage” research project and by NODES (“Nord Ovest Digitale e Sostenibile”) project, which has received funding from the MUR – M4C2 1.5 of PNRR with grant agreement n. ECS00000036.

## Conflict of interest

The authors declare that the research was conducted in the absence of any commercial or financial relationships that could be construed as a potential conflict of interest.

## Publisher’s note

All claims expressed in this article are solely those of the authors and do not necessarily represent those of their affiliated organizations, or those of the publisher, the editors and the reviewers. Any product that may be evaluated in this article, or claim that may be made by its manufacturer, is not guaranteed or endorsed by the publisher.
